# Supplementary material for: MetaRibo-Seq measures translation in microbiomes
Source: Nat Commun. 2020 Jun 29;11:3268. doi: 10.1038/s41467-020-17081-z (PMC7324362; doi:10.1038/s41467-020-17081-z)
Supplement: Supplementary file 10 — Supplementary Data 7 [file 41467_2020_17081_MOESM10_ESM.zip › File2/Confidence_High_Taxonomy/333373_out.krona.html]

Javascript must be enabled to view this page.

members
magnitude
magnitudeUnassigned
count
unassigned
taxon
rank

333373\_out

50

superkingdom
2
50

1239
50
phylum

species

SRS075878\_contig\_number\_18820
1263002
1

186801
49
class

186802
49
order

49
541000
family

2
1263
genus

species
1262952

SRS014412\_contig\_number\_13679SRS049773\_contig\_number\_28336
2

1
216851
genus

species
853

SRS050998\_contig\_number\_13020
1

46
1898205

SRS012273\_contig\_number\_31394SRS012849\_contig\_number\_25153SRS013098\_contig\_number\_29213SRS013476\_contig\_number\_contig-100\_3510.193620SRS014235\_contig\_number\_34174SRS015431\_contig\_number\_32677SRS015782\_contig\_number\_35779SRS016629\_contig\_number\_3464SRS017103\_contig\_number\_contig-100\_388.180692SRS018575\_contig\_number\_contig-100\_7688.36711SRS018623\_contig\_number\_13161SRS018817\_contig\_number\_11397SRS018836\_contig\_number\_24576SRS019068\_contig\_number\_51929SRS019601\_contig\_number\_contig-100\_1617.137163SRS019910\_contig\_number\_contig-100\_4620.121955SRS020869\_contig\_number\_32278SRS023914\_contig\_number\_15873SRS024331\_contig\_number\_36084SRS024435\_contig\_number\_31973SRS042966\_contig\_number\_19081SRS047433\_contig\_number\_13987SRS049446\_contig\_number\_10352SRS049896\_contig\_number\_24909SRS049959\_contig\_number\_6454SRS050752\_contig\_number\_19887SRS053356\_contig\_number\_1548SRS097920\_contig\_number\_14172SRS098571\_contig\_number\_46510SRS1041032\_contig\_number\_14393SRS1041039\_contig\_number\_19706SRS1041137\_contig\_number\_12453SRS1041157\_contig\_number\_5394SRS1055056\_contig\_number\_574SRS143417\_contig\_number\_7291SRS143895\_contig\_number\_17095SRS144362\_contig\_number\_17735SRS144537\_contig\_number\_18423SRS147557\_contig\_number\_27184SRS148196\_contig\_number\_17881SRS149325\_contig\_number\_12244SRS149784\_contig\_number\_14153SRS893279\_contig\_number\_5269SRS893369\_contig\_number\_9310SRS893373\_contig\_number\_4401SRS971275\_contig\_number\_30762
species
